# Blood Biomarker Tenascin-C for Detection of Radiation Cardiotoxicity: An Animal Model

**DOI:** 10.3390/jcm15145724

**Published:** 2026-07-21

**Authors:** Ecem Demir, Karolin Yanar, Pınar Atukeren, Serbay Ozkan, Gözde Erkanlı Şentürk, Melike Ülker, Şefika Arzu Ergen, Songül Karaçam, Fazilet Öner Dinçbaş

**Affiliations:** 1 Department of Radiation Oncology Istanbul, Başakşehir Çam and Sakura City Hospital, Istanbul 34480, Turkey; 2 Department of Biochemistry, Cerrahpaşa Faculty of Medicine, Istanbul University-Cerrahpaşa, Istanbul 34320, Turkey; 3 Department of Histology and Embryology, Faculty of Medicine, Izmir Katipcelebi University, Izmir 35620, Turkey; 4 Department of Histology and Embryology, Cerrahpaşa Faculty of Medicine, Istanbul University-Cerrahpaşa, Istanbul 34098, Turkey; 5 Department of Thoracic Surgery, Avrasya Hospital, Istanbul 34020, Turkey; 6 Department of Radiation Oncology Istanbul, Cerrahpaşa Faculty of Medicine, Istanbul University-Cerrahpaşa, Istanbul 34098, Turkey; 7 Radiotherapy Program, Vocational School of Health Services, Istanbul University-Cerrahpaşa, Istanbul 34098, Turkey

**Keywords:** radiation injuries, cardiotoxicity, tenascin-C, radioprotection

## Abstract

**Purpose:** The aim of this study was to test the hypothesis that Tenascin-C (TNC) levels increase following thoracic radiotherapy (RT), and that elevated serum TNC levels are associated with corresponding increases in myocardial TNC expression in histopathological specimens, supporting its role as a potential biomarker of radiation-induced cardiotoxicity. **Methods:** Blood and cardiac tissue samples from rats 8 weeks after thoracic radiotherapy were analyzed to evaluate changes in serum TNC levels and myocardial TNC expression, and their association with radiation exposure. Male Wistar rats were randomized to a single 12-Gy dose of mediastinal irradiation or sham irradiation. Serum TNC levels were assessed with rat ELISA kits. Myocardial TNC expression was assessed using immunohistochemistry, and staining intensity was semi-quantitatively graded by blinded observers. **Results:** Serum Tenascin-C (TNC) levels were elevated in RT only group compared to control (*p* < 0.001), paralleled by a marked increase in myocardial TNC expression (*p* < 0.001). A consistent concordance between circulating and tissue-level TNC was observed, supporting its potential as a biomarker of radiation-induced cardiac injury. **Conclusions:** Significant associations were observed between radiation exposure and both serum Tenascin-C levels and myocardial Tenascin-C expression, demonstrating a concordance between circulating and tissue findings and supporting Tenascin-C as a promising biomarker of radiation-induced cardiotoxicity.

## 1. Introduction

Thoracic radiotherapy (RT) is a key component of curative treatment for lung cancer. During the delivery of curative radiation doses to thoracic tumors, the heart may receive incidental exposure from the treatment field. This exposure can contribute to radiation-induced cardiotoxicity, which is often recognized only after cardiac injury has progressed to an irreversible stage [1]. The underlying biological mechanisms and the chronological course of radiation-induced cardiac injury remain incompletely understood [2]. Conventional diagnostic tools such as echocardiography and electrocardiography frequently detect abnormalities only in later stages, and patients may remain asymptomatic despite ongoing subclinical ischemic events [3]. Therefore, there is a need for potential biomarkers that can identify radiation-related cardiac injury before irreversible functional impairment occurs.

The acute effects of radiation are primarily mediated through reactive oxygen species and activation of TNF-α, IL-1, and IL-6 [4]. These mediators start intracellular signaling and myocardial tissue responses with edema, vascular injury, increased permeability, and vasodilation [5]. Persistent inflammation promotes fibroblast activation, leading to increased collagen and extracellular matrix protein synthesis. Over time, these responses manifest as accelerated atherosclerosis. Also, profibrotic growth factors such as transforming growth factor-beta (TGF-B) are upregulated, which increases fibrotic remodeling [6]. Continuous oxidative stress, microvascular damage, and stem cell depletion ultimately result in myocardial fibrosis, which ends with morbid late complications such as chronic heart failure and myocardial infarction [7].

In recent years, several biomarkers have been proposed for the detection of radiation cardiotoxicity, such as pro-BNP, troponin T [8]. Among these, Tenascin-C (TNC) has emerged as a promising candidate due to its cardiac-specific expression and its role in inflammation and extracellular matrix remodeling [9]. TNC has been shown to serve as a diagnostic and prognostic biomarker in various inflammatory cardiac conditions and may also be utilized as a molecular imaging target [10]. However, to date, there is no accessible and indexed literature specifically evaluating the role of TNC in radiation cardiotoxicity. The aim of the present study was to investigate the effectiveness of TNC as a potential biomarker for detecting cardiotoxicity following mediastinal.

## 2. Materials and Methods

This study was approved by the Bezmialem University Ethics Committee (Approval No: 05.05.2025-E.191908).

### 2.1. Animals

Eighteen adult male Sprague-Dawley rats were included in the study. Male rats (200–300 g) were preferred based on prior evidence indicating sex-related differences in radiation response, particularly in cardiac and pulmonary injury models [11]. Animals were housed under standardized conditions (23 ± 2 °C, 55 ± 10% humidity, 12-h light/dark cycle) and were fed standard rat chow and water ad libitum. Following acclimatization, animals were randomly divided into RT or control group. The ARRIVE Checklist for Animal Study can be found in Supplementary Table S1.

### 2.2. Radiotherapy Protocol

Animals were anesthetized with intramuscular ketamine (100 mg/kg) and xylazine (10 mg/kg), then positioned supine and immobilized for computed tomography (CT) simulation.

**Figure 1:**
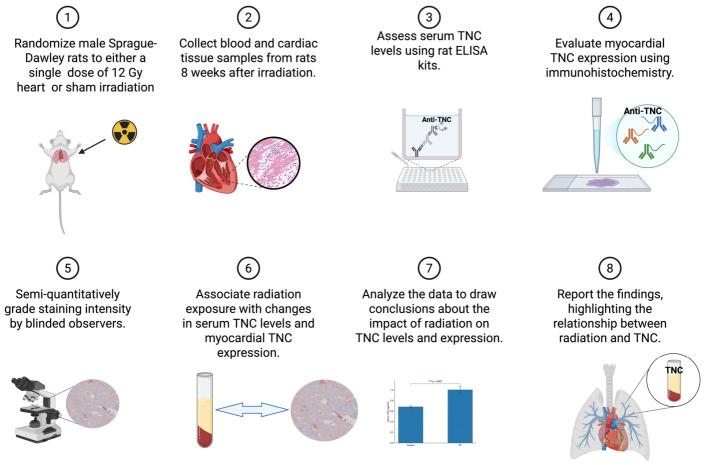
Experimental design of radiation-induced cardiotoxicity model and assessment of serum and myocardial Tenascin-C expression. Created with BioRender.com by the author (Ecem Demir, 2026), URL: https://BioRender.com/ouovz2b (accessed on 12 July 2026).

The whole heart volume was contoured on CT images, and a 3D conformal RT (3D-CRT) treatment plan was made. The rats were then irradiated with an X-ray linear accelerator device (Rapid Arc, Varian Medical Systems, Palo Alto, CA, USA) using a 6 megavolt (MV) photon beam at 100 cm from the source to the surface. The whole heart volume received 12 Gy X-rays in a single fraction at a dose rate of 300 monitor units (MU). A 1 cm thick elastic-gel bolus was placed 1 cm above the thorax to maximise the cardiac dose [12]. A single 12-Gy cardiac irradiation dose was selected based on our previously established radiation-induced cardiotoxicity model, in which this dose produced measurable oxidative stress alterations and histopathological cardiac injury at 8 weeks while maintaining animal survival until the planned endpoint [12]. The experimental protocol is given in Figure 1.

### 2.3. Collection of Rat Serum and TNC Biochemical Evaluation

The experimental duration was set at 8 weeks, corresponding to the timeframe for subacute radiation-induced cardiac injury [13,14]. The 8-week post-irradiation time point was selected based on our previously established radiation-induced cardiotoxicity animal model, in which cardiac irradiation was planned using simulation CT, the whole heart was contoured as the target volume, and a single 12-Gy fraction was delivered to the cardiac target. In that model, biochemical oxidative stress alterations and histopathological cardiac injury were assessed 8 weeks after irradiation, supporting this interval as an early/subacute phase suitable for evaluating radiation-induced cardiac remodeling. The present study was designed as a secondary biomarker-focused extension of this experimental framework, aiming to investigate whether serum and myocardial Tenascin-C expression are increased at this biologically relevant post-irradiation interval [12].

At the end of the study, animals were euthanized under anesthesia via exsanguination, and cardiac tissues were harvested for histopathological analysis. At the 8th week, the animals were euthanized by exsanguination following anesthesia. Blood sampling was performed without fasting, with samples collected via cardiac puncture. Blood was collected into heparinised collection tubes containing EDTA and serum tubes. Serum and plasma samples obtained by centrifugation at 2000 rpm for 10 min using a chilled centrifuge were pipetted into Eppendorf tubes and stored at −20 °C until evaluation in 1 month.

A commercially available sandwich-type ELISA kit (ELK, Wuhan, China) was used for analysis. The ELISA plate was pre-coated with an antibody specific for rat TNC. Rat serum samples were added to the wells, followed by incubation and washing steps. Subsequently, a biotin-conjugated antibody specific for rat TNC was added and incubated, followed by another washing step. Horseradish peroxidase (HRP)-conjugated avidin was then added and incubated. After additional washing, a tetramethylbenzidine (TMB) substrate solution was added, resulting in the development of a blue color. The reaction was terminated by adding a stop solution containing sulfuric acid, which changed the color to yellow. The absorbance was measured at 450 nm, and TNC concentrations were calculated from a standard calibration curve generated using standard solutions. Results were expressed in ng/mL.

### 2.4. Histopathological Evaluation

Formalin-fixed, paraffin-embedded cardiac tissues were analyzed for Tenascin-C expression using a rat-specific immunohistochemistry kit (IHCeasy TNC/Tenascin-C Ready-to-use IHC Kit, Proteintech, KCH0113, Rosemont, IL, USA). Staining intensity was evaluated semi-quantitatively.

Myocardial Tenascin-C (TNC) immunoreactivity was evaluated by immunohistochemistry using a semi-quantitative scoring system based on staining intensity. Immunoreactivity was graded on a scale from 0 to 3 (0 = no immunoreactivity, 1 = mild, 2 = moderate, 3 = severe). Scoring was performed by blinded observers using light microscopy, focusing on extracellular matrix and perivascular staining patterns. Individual scores were recorded for each specimen, and group-level differences were analyzed using non-parametric statistical methods.

### 2.5. Statistical Analysis

Biochemical parameters of the treatment and control groups were compared using the Mann–Whitney U test. All data are presented as mean ± standard deviation (SD) and median. A *p*-value <0.05 was considered statistically significant. Statistical analyses were performed using SPSS v29. Bonferroni correction was used for multiple comparisons.

To directly assess the relationship between circulating and tissue-level TNC, Spearman’s rank correlation analysis was performed between individual serum TNC concentrations and corresponding myocardial TNC immunohistochemical scores across all animals.

## 3. Results

### 3.1. TNC Expression in Irradiated Versus Control Myocardium

**Figure 2:**
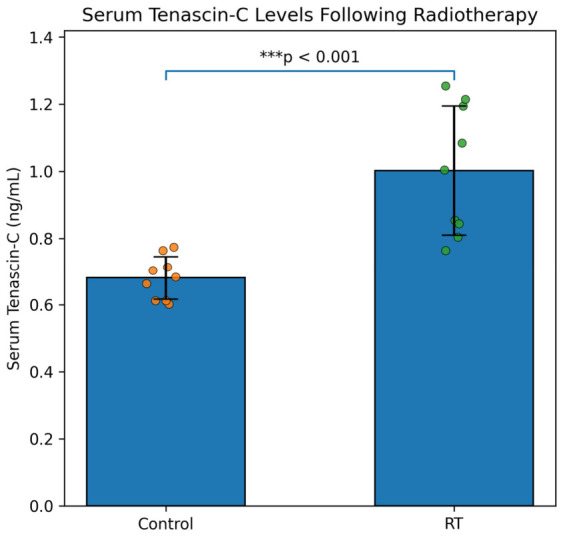
Increased Serum Tenascin-C Levels Following Radiotherapy. Serum Tenascin-C (TNC) levels in control and radiotherapy (RT) groups. Data are expressed as mean ± SD, with individual animal values shown as overlaid points. A significant increase in TNC levels was observed in the RT group compared with controls (*** *p* < 0.001, Mann–Whitney U test), indicating an association between radiation exposure and elevated circulating TNC levels.

A total of 18 animals were analyzed, including 9 in the RT group and 9 in the control group. Serum Tenascin-C (TNC) levels were significantly elevated in the RT group compared with controls (1.00 ± 0.19 vs. 0.68 ± 0.06). This difference was confirmed by Mann–Whitney U analysis (U = 1.5, Z = −3.447, *p* < 0.001), demonstrating a strong association between radiation exposure and increased circulating TNC levels, shown in Figure 2.

### 3.2. Morphological Observations

**Figure 3:**
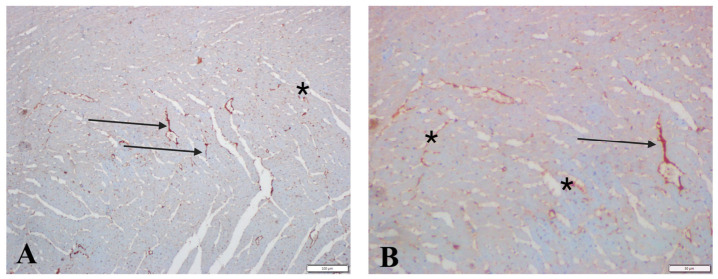
Immunohistochemical evaluation of myocardial tenascin-C expression following irradiation. Immunohistochemical staining was performed using a ready-to-use Tenascin-C IHC kit (Proteintech, KCH0113) according to the manufacturer’s instructions. Detection was carried out using an HRP-based system, and sections were counterstained with hematoxylin. (**A**) Non-irradiated control group showing minimal basal TNC expression within the myocardial extracellular matrix. (**B**) Irradiated group demonstrating increased TNC immunoreactivity, predominantly localized in interstitial and perivascular regions, with a reticular/fibrillar staining pattern consistent with early extracellular matrix remodeling and inflammatory activation. TNC immunoreactivity was mainly observed within interstitial and perivascular compartments. Arrows indicate interstitial and perivascular Tenascin-C expression; asterisks (*) denote areas of increased immunoreactivity. Scale bars: (**A**) 100 μm; (**B**) 50 μm. Created with BioRender.com by the author (Ecem Demir, 2026), https://BioRender.com/ouovz2b (accessed on 12 July 2026).

Semi-quantitative immunohistochemical evaluation revealed significantly higher myocardial Tenascin-C (TNC) expression in the RT group compared with controls (2.17 ± 0.35 vs. 1.11 ± 0.42). This difference was confirmed by Mann–Whitney U analysis (U = 3.5, Z = −3.488, *p* < 0.001), with substantially higher mean ranks in the RT group (13.61 vs. 5.39), supporting increased extracellular matrix remodeling following irradiation (Figure 3).

### 3.3. Exploratory Relationship Between Circulating and Tissue-Level TNC

**Figure 4:**
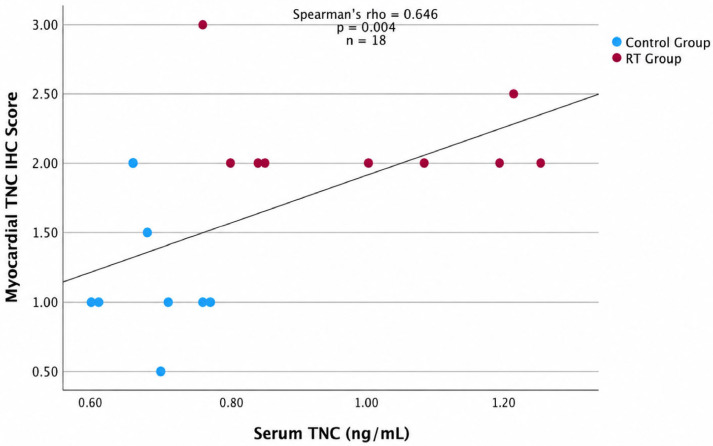
Correlation between serum Tenascin-C levels and myocardial Tenascin-C immunohistochemical scores.

To directly evaluate the relationship between circulating and myocardial TNC, individual serum TNC concentrations were correlated with the corresponding myocardial immunohistochemical scores. An exploratory Spearman rank correlation analysis demonstrated a significant positive association between serum TNC levels and myocardial TNC expression scores across all animals (Spearman’s rho = 0.646, *p* = 0.0038, *n* = 18), indicating that higher circulating TNC levels were associated with stronger myocardial TNC immunoreactivity (Figure 4).

## 4. Discussion

Radiation-induced cardiac injury is primarily mediated by endothelial dysfunction, oxidative stress, and progressive myocardial remodeling. Ionizing radiation induces direct DNA damage and generates reactive oxygen species (ROS), leading to cellular stress, apoptosis, and activation of pro-inflammatory signaling pathways [15]. Endothelial injury represents a central initiating event, resulting in increased vascular permeability, leukocyte adhesion, and a pro-thrombotic and pro-inflammatory microenvironment that promotes fibrosis and tissue degeneration [16]. In parallel, radiation-induced upregulation of inflammatory cytokines, including IL-6, IL-8, and TNF-α, further amplifies inflammatory signaling and contributes to extracellular matrix remodeling and fibroblast activation [17]. Microvascular damage and impaired perfusion progressively lead to ischemia and fibrotic transformation of the myocardium, which constitute the hallmark features of radiation-induced heart disease [18].

While established biomarkers such as cardiac troponins and natriuretic peptides have been widely used for the detection of cardiotoxicity, particularly in chemotherapy settings, their role in radiation-induced cardiac injury remains limited and inconsistent. Emerging evidence suggests that several novel biomarkers—including myeloperoxidase, arginine–nitric oxide metabolites, and growth differentiation factor-15—may provide additional mechanistic insight into early cardiac injury; however, these markers still require further validation before clinical implementation [19]. Furthermore, biomarkers reflecting vascular endothelial function, such as placental growth factor, have been proposed as potential indicators of radiation-induced cardiac damage, although their clinical applicability remains uncertain [20].

In the present study, TNC emerged as a sensitive marker of myocardial injury following radiation exposure. Serum TNC levels were significantly elevated in the radiotherapy group and paralleled by increased myocardial TNC expression on immunohistochemical analysis, supporting a close association between circulating levels and tissue-level remodeling. This finding is consistent with the known induction of TNC by inflammatory cytokines, oxidative stress, and mechanical injury—key drivers of radiation-induced cardiac damage.

Mechanistically, TNC upregulation may reflect early fibroblast activation and extracellular matrix remodeling, along with amplification of inflammatory signaling within interstitial and perivascular compartments. The concordance between serum and tissue findings suggests that circulating TNC may serve as a non-invasive surrogate of myocardial injury. Nevertheless, whether TNC represents a mediator of radiation-induced damage or a downstream response remains unclear, warranting further investigation.

Although myocardial immunohistochemistry demonstrated increased cardiac TNC expression following irradiation, the possibility of a non-cardiac contribution to circulating TNC should be acknowledged. In the present model, RT was planned on simulation CT images, the whole heart was specifically contoured as the target volume, and a single 12-Gy dose was delivered to the cardiac target. Therefore, the observed increase in serum TNC is likely to predominantly reflect radiation-induced cardiac injury, which is also supported by the increased myocardial TNC immunoreactivity. But, the treatment field was located within the thorax, and adjacent lung tissue may have been exposed to a low-dose radiation bath, and TNC may also be induced during pulmonary inflammatory and fibrotic remodeling. Since pulmonary histopathological TNC evaluation was not performed, a minor pulmonary contribution to circulating TNC cannot be completely excluded. Accordingly, TNC should be interpreted as a candidate biomarker associated with radiation-induced cardiac remodeling in this preclinical model, rather than as a validated clinical biomarker of radiation-induced cardiotoxicity. Future studies, including parallel cardiac and pulmonary histopathological assessment and organ-specific dose–response analyses, are warranted to clarify the relative tissue contributions to circulating TNC.

To the best of our knowledge, this is the first study to demonstrate the potential utility of Tenascin-C as a biomarker of radiation-induced cardiac injury. Several limitations should be acknowledged. The study was conducted in a preclinical model with a relatively small sample size, and functional cardiac assessments were not included. Circulating TNC levels may be influenced by systemic factors, although the concordant tissue findings strengthen the specificity of our results. The use of a single 8-week endpoint remains an important limitation. Earlier and later time points would be required to define whether TNC can be considered an early biomarker. Also, the use of a single 12-Gy fraction represents an experimental injury model and does not directly replicate conventional clinical fractionated thoracic RT. In addition, the correlation analysis was exploratory, involved only 18 animals, and pooled irradiated and control groups. Therefore, the observed association may partly reflect treatment-group separation and should not be interpreted as definitive evidence that circulating TNC is a validated surrogate of myocardial TNC expression.

## 5. Conclusions

An exploratory positive correlation was observed between circulating TNC concentrations and myocardial immunohistochemical scores at 8 weeks after targeted cardiac irradiation, and a significant positive correlation was observed, suggesting that TNC may represent a candidate biomarker associated with subacute radiation-induced cardiac remodeling. However, further studies incorporating serial time points, functional cardiac assessment, and clinically relevant fractionated irradiation schedules are required before TNC can be considered a validated biomarker of radiation-induced cardiotoxicity.

## Data Availability

Data are available from the corresponding author upon reasonable request, as no publicly archived dataset was generated for this animal study.

## Supplementary Materials

The following supporting information can be downloaded at: https://www.mdpi.com/article/10.3390/jcm15145724/s1, Table S1: The ARRIVE Checklist for Animal Study.
